# Mechanisms underlying the protective effects of mesenchymal stem cell-based therapy

**DOI:** 10.1007/s00018-020-03454-6

**Published:** 2020-01-21

**Authors:** Xing-Liang Fan, Yuelin Zhang, Xin Li, Qing-Ling Fu

**Affiliations:** 1 Otorhinolaryngology Hospital, The First Affiliated Hospital, Sun Yat-Sen University, 58 Zhongshan Road II, Guangzhou, 510080 People’s Republic of China; 2 Department of Emergency, Guangdong Provincial People’s Hospital, Guangdong Academy of Medical Sciences, 106 Zhongshan Road II, Guangzhou, 510080 People’s Republic of China; 3 Key Laboratory for Stem Cells and Tissue Engineering, Ministry of Education, Sun Yat-Sen University, Guangzhou, Guangdong, People’s Republic of China

**Keywords:** Regenerative potential, Integration of MSCs, Immunomodulation, Soluble factors, Cell–cell contact, Mitochondrial transfer, Extracellular vesicles

## Abstract

Mesenchymal stem cells (MSCs) have been extensively investigated for the treatment of various diseases. The therapeutic potential of MSCs is attributed to complex cellular and molecular mechanisms of action including differentiation into multiple cell lineages and regulation of immune responses via immunomodulation. The plasticity of MSCs in immunomodulation allow these cells to exert different immune effects depending on different diseases. Understanding the biology of MSCs and their role in treatment is critical to determine their potential for various therapeutic applications and for the development of MSC-based regenerative medicine. This review summarizes the recent progress of particular mechanisms underlying the tissue regenerative properties and immunomodulatory effects of MSCs. We focused on discussing the functional roles of paracrine activities, direct cell–cell contact, mitochondrial transfer, and extracellular vesicles related to MSC-mediated effects on immune cell responses, cell survival, and regeneration. This will provide an overview of the current research on the rapid development of MSC-based therapies.

## Introduction

Mesenchymal stem cells (MSCs), alternatively referred to as mesenchymal stromal cells, have been extensively investigated since their discovery in the bone marrow by Alexander Friedenstein and colleagues in the late 1960s [1, 2]. MSCs can migrate to injured sites, engraft, and differentiate into end-stage functional cells, thus repairing the injured tissue [3, 4]. More importantly, MSCs have also shown promising therapeutic effects due to their ability to modulate multiple immune cell types of both the innate and adaptive immune systems. MSCs can promote neovascularization, increase angiogenesis, enhance cell viability and/or proliferation, inhibit cell death, and modulate immune responses via paracrine and cell–cell contact effects as well as through extracellular vesicles [5, 6]. Recently, over 900 clinical trials worldwide have used MSCs to treat various diseases (www.clinicaltrials.gov), including bone/cartilage repair, diabetes, cardiovascular diseases, immune-related, and neurological disorders. MSCs are attractive candidates for treating various diseases because they can travel to injured sites, differentiate into multiple cell types, and regulate immunomodulation [7]. In particular, the role of homing in MSC-based therapies remains doubtful. Interestingly, despite some encouraging results from animal studies, some clinical trials have also shown no therapeutic efficacy of MSCs. Therefore, understanding the biology of MSCs and their role in treatment will be critical to determine their potential for various therapeutic applications. This review summarizes the mechanisms underlying the protective effects of MSCs and provides an overview of the recent developments in MSC-based therapy.

### MSC identity

MSCs are classically defined as plastic-adherent, expanding, non-hematopoietic cells that can differentiate into osteoblasts (bone cells), adipocytes (fat cells), chondroblasts (cartilage cells) and myocytes (skeletal muscle cells) in vitro [8–10]. They express the cluster of differentiation (CD) surface markers including CD90, CD105, and CD73, but do not express CD11b, CD14, CD19, CD34, CD45 and human leukocyte antigen (HLA)-DR according to the International Society of Cell Therapy (ISCT) criteria [8, 9, 11]. However, this set of cell surface markers is not always applicable when identifying MSCs as pericytes and defining the cell markers. MSCs isolated from different tissues have different surface antigen molecules because these surface markers are influenced by many factors. The surface markers of MSCs isolated from the lung are distinct from those of the MSCs derived from the bone marrow [12]. Additional/alternative markers are being identified and confirmed for some specific sources of MSCs. For instance, CD146 is essential for MSC vigour and self-renewal as the dividing ability of MSCs is weakened or eliminated when *CD146* is downregulated or silenced [13]. CD49d is detected in adipose-derived MSCs but not in BM-MSCs [14]. To date, markers for identification of MSCs are under investigation. There needs to be a more critical take on a field that has deviated from careful science.

### Sources of MSCs

**Fig. 1:**
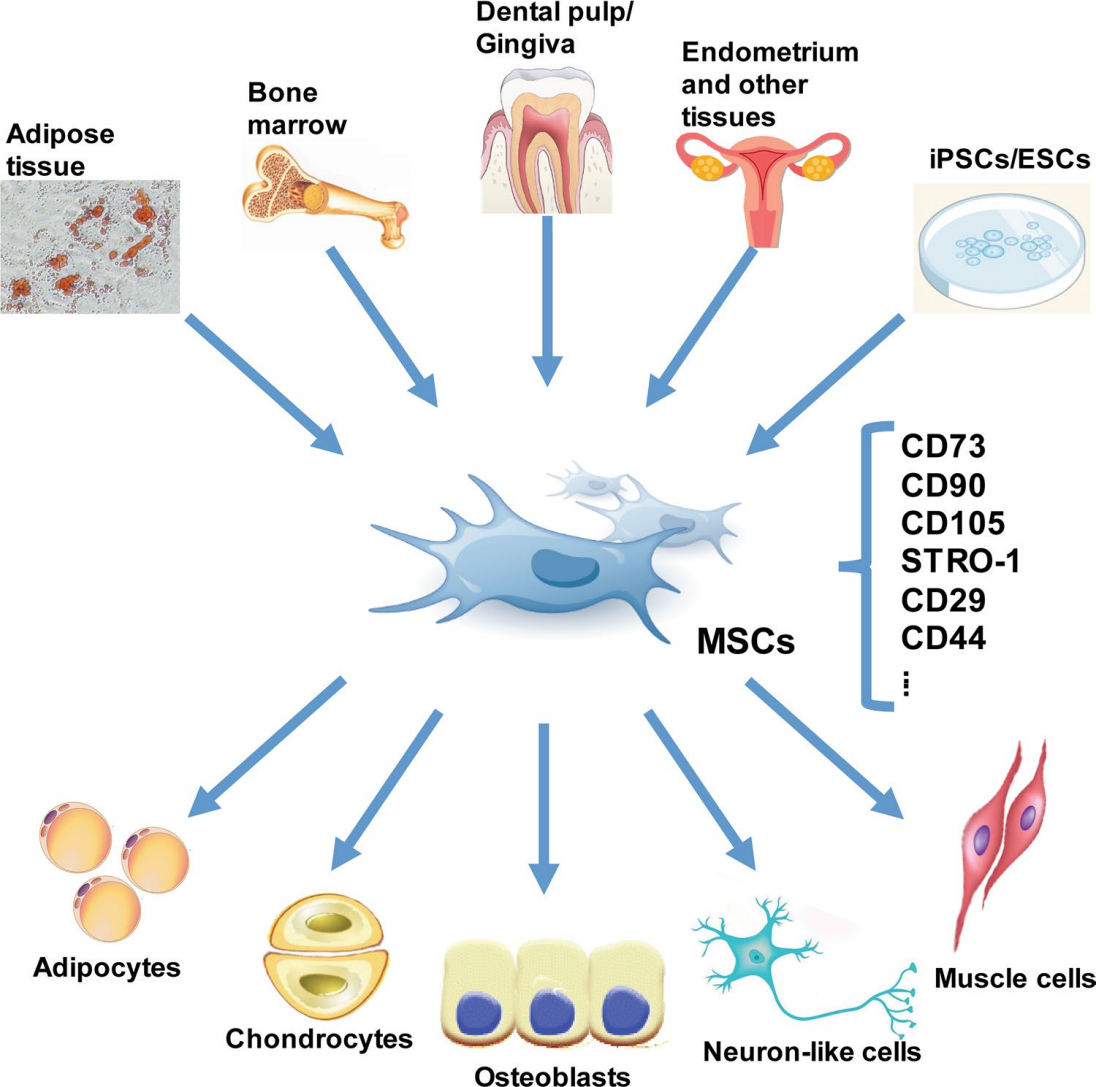
MSCs can be isolated from a variety of foetal, neonatal, and adult tissues, and can differentiate into different cell types. *CD* cluster of differentiation, *ESCs* embryonic stem cells, *iPSCs* induced pluripotent stem cells, *MSCs* mesenchymal stem cells

Although bone marrow is the conventional source of MSCs, MSCs or MSC-like cells can be isolated from almost any tissue of the human body. MSC-like cells have been isolated from a variety of foetal, neonatal, and adult tissues including adipose tissue, amniotic fluid, brain, compact bone, dermis, dental pulp, gingiva, foetal liver and lung, human islets, placenta, skeletal muscle, synovium, umbilical cord, peripheral blood and so on (Fig. 1) [14–24]. It is considered that MSCs refer to cells derived from the bone marrow, but not necessarily those from other sites such as adipose tissue, which are often termed as adipose-derived stem cells (ASCs). However, MSCs derived from different origins have different characteristics and differentiation potential [25, 26]. Moreover, MSCs from different sources display significant differences in the levels of several paracrine factors [27]. Currently, the most frequently reported sources of MSCs utilized in clinical trials are the bone marrow, adipose tissue, and umbilical cord. This is partially due to the accessibility, ease of isolation, and MSC-based repair efficacy. The characteristics and differentiation potential of the most commonly investigated MSCs derived from different tissues have been summarized in Table 1.

**Table 1:** Characteristics and differentiation potential of the common different tissue-derived MSCs

Source tissue	Characteristics	Differentiation potential	References
Adipose tissue	CD73, CD90, CD29, CD44, CD71, CD105, CD13, CD166, STRO-1	Adipocyte, chondrocyte, osteoblast	[29–31]
Amniotic fluid	CD44, CD90, CD105, CD13, CD29, CD71, CD120a	Adipocyte, cardiomyocyte-like cell, chondrocyte, osteoblast	[32, 33]
Bone marrow	CD73, CD90, CD105, STRO-1	Adipocyte, chondrocyte, osteoblast, tenocyte, vascular smooth muscle cell	[34–37]
Dental pulp	CD29, CD44, CD90, CD105	Adipocyte, chondrocyte, osteoblast, neuron-like cell, odontoblast, myogenic lineages	[38–41]
Endometrium	CD29, CD90, CD73, CD105	Adipocyte, chondrocyte, osteoblast	[42, 43]
Peripheral blood	CD44, CD90, CD105, HLA-ABC	Adipocyte, osteoblast, fibroblast	[44]
Placenta	CD29, CD73, CD90, CD105	Adipocyte, chondrocyte, osteoblast, myotubular cell, pancreatic progenitor cell, neuron-like cell, retinal cell	[45, 46]
Synovium	CD44, CD90, CD105, CD147, STRO-1	Adipocyte, chondrocyte, osteoblast, skeletal muscle cell	[29, 47, 48]
Skin	CD44, CD73, CD90, CD105, CD166, SSEA-4, Vimentin	Adipocyte, chondrocyte, osteoblast, neuron-like cell, pancreatic cell, endothelial cell	[49–51]
Umbilical cord	CD29, CD44, CD73, CD90, CD105	Adipocyte, chondrocyte, osteoblast, skeletal muscle cell, endothelial cell, cardiomyocyte-like cell, neuron-like cell	[52, 53]

Although MSCs can hypothetically be obtained from almost any tissue within the human body, the MSC surface markers, quality and isolated numbers are restricted by various donor characteristics. There are also practical limitations concerning the difficulty and invasiveness of the procurement process [25]. To select an adequate cell source, the practitioner must consider both advantages and disadvantages of procuring MSCs with regard to the difficulty and potential adverse effects of harvesting donor the cells. For instance, BM-MSCs have shown confirmed safety and effectiveness in multiple clinical trials, but their yields and differentiation potential are dependent on the donor characteristics (e.g., age). Moreover, isolation of cells from the bone marrow is often painful and carries the risk of infection. As adipose tissue is accessible and abundant, this source results in the isolation of stem cells that is 500 times more than the ones obtained from the bone marrow. Adipose tissue-derived MSCs have stronger immunosuppressive effects but have inferior osteogenic and chondrogenic potential as compared to the potential seen in BM-MSCs. The frequency of colony-forming cells from dental pulp is high compared to those from bone marrow, and the source materials are easily accessible as dental surgeries are fairly common. However, ectomesenchymal and periodontal tissues can affect the properties of dental pulp-derived MSCs [28]. MSCs derived from birth-related tissues (amnion, placenta and umbilical cord) demonstrate higher expansion and engraftment capacity, but these cells are not as useful as those from bone marrow or blood in terms of osteogenesis [25]. Obtaining MSCs from different tissues will demonstrate various characteristics that may differ due to the tissue source, health condition, and age of the donor. Thus, researchers have begun to differentiate MSCs from pluripotent stem cells, to circumvent the drawbacks of tissue-derived MSCs.

MSCs can be derived from pluripotent stem cells including human embryonic stem cells (hESCs) and induced pluripotent stem cells (iPSCs) [54, 55]. Despite no direct sequencing comparisons between pluripotent stem cell-derived MSCs and BM-MSCs, iPSC-MSCs indeed express typical MSC surface markers and undergo adipogenesis, osteogenesis, and chondrogenesis similar to that observed in adult BM-MSCs [55–57]. The functional characteristics of iPSC-MSCs have made the cells usable for tissue engineering and cellular therapeutics. More importantly, MSCs derived from pluripotent stem cells display a higher proliferative capacity and telomerase activity. These cells have a higher proliferative capacity (more than 50 passages), and lower cell senescence than that observed in BM-MSCs [55, 57, 58]. We also observed no teratogenic effects of iPSC-MSCs in animal studies, implying the safety of using iPSC-MSCs [55]. Furthermore, iPSC-MSCs from aged individuals were reported acquire a rejuvenation signature, which circumvents the ageing-associated drawbacks [59]. A very large number of functional MSCs can be clonally generated from a single-cell level, which maintains the homogeneity and functional quality of MSCs.

Moreover, compared with BM-MSCs, iPSC-MSCs are more insensitive to pro-inflammatory interferon (IFN)-γ-induced HLA-II expression, exhibiting stronger immune privilege, superior survival rates, and improved engraftment after transplantation. This means that pluripotent stem cell-derived MSCs have a stronger advantage in allogeneic transplantation [58]. Recently, the use of adult tissues, especially bone marrow, as a source of MSCs has decreased [60]. However, pluripotent stem cell-derived MSC-based therapy is in the early investigational stage and is not ready for clinical application as many challenges remain to be overcome. For instance, there are ethical issues in hESC application. Will the original tissue/cell of iPSCs ultimately affect the function and effect of MSCs in different diseases? Will iPSC reprogramming cause genome instability? Genome sequencing should thus be carried out to verify the correctness of each base in iPSCs. Direct comparisons using advanced techniques such as RNAseq indicating that pluripotent stem cell-derived MSCs are similar to MSCs derived from adult tissues will be favourable. The reprogramming method may also affect the function of MSCs, but existing reprogramming methods such as those using small molecular compounds can avoid the hidden dangers posed by virus-mediated reprogramming.

### MSC functions

In addition to cells of the mesodermal lineage (i.e., adipocytes, chondrocytes, osteoblasts and skeletal myocytes), MSCs also can differentiate into cells of ectodermal origin and endodermal origin, such as hepatocytes and neuron-like cells [61, 62]. Significant progress has been achieved in tissue regeneration using MSCs in de-cellularized or synthetic scaffolds [63]. However, the encouraging regenerative potential of MSCs is mainly validated by in vitro functional assays. Upon allogeneic transplantation, MSCs have shown protective effects in a variety of injured models including damaged bone, cartilage [64], hepatic [65], myocardial [66], and neural tissues [67]. However, the therapeutic effects of MSCs are not attributed to poor cell retention [68].

It is becoming increasingly evident that the therapeutic effects of MSCs are largely attributed to the immunomodulatory function. MSCs exert immunomodulatory and anti-inflammatory effects by regulating lymphocytes associated with both innate and adaptive immune systems [68]. It is well documented that MSCs regulate the immune response in many diseases [69]. Accumulating evidences have demonstrated that MSCs can regulate T cell proliferation, function, balance T-helper (Th)1 and Th2 activity [69, 70], upregulate the functions of regulatory T cells (Tregs) [71], suppress B cell functions [72, 73], inhibit natural killer (NK) cell proliferation and function [74], and prevent dendritic cell (DC) maturation and activation [6, 57, 62]. MSCs can also stimulate proliferation and cytokine secretion in innate lymphoid cells (ILCs), a new family of lymphocyte-like cells, which play an important role in innate defences against pathogens [75, 76]. MSCs can regulate their immunomodulatory functions according to the micro-environmental inflammatory conditions. The plasticity of MSCs in immunomodulation is affected by the type and intensity of inflammatory stimuli conferred on MSCs. For instance, MSCs can suppress the polarization of Th1 and Th17, and promote Th2 polarization in graft-versus-host disease (GvHD) [77]. Meanwhile, MSCs can also inhibit Th2-dominant allergy by inhibiting IL-4 and IL-13 production [7]. Furthermore, MSCs promote the responses of lymphocytes in quiescent-state peripheral blood mononuclear cells (PBMCs) from patients with allergic rhinitis [70]. MSCs exert immunosuppressive effects or contribute to the fibrotic process under acute or chronic inflammatory conditions, respectively [62, 78]. Their immunomodulatory characteristics thus make MSCs a flexible and feasible strategy for treating various diseases.

The function of MSCs is known to decline with age, a process that may be implicated in the loss of tissue homeostasis leading to organ failure and aging-related diseases [79]. The proliferative and functional activity of MSCs is destined to decline during the process of senescence. The osteogenic activity of senescent MSCs deteriorates as a function of increasing lifespan, whereas the adipogenic differentiation potential of MSCs remains unchanged or is even enhanced [80]. For osteogenic induction, early passages MSCs or strategies to prevent senescence must be considered to yield longer osteogenesis and better quality. Furthermore, the immunomodulatory functions of MSCs are also reported to be compromised due to increased reactive oxygen species and oxidative stress in aged cells [81]. Therefore, MSC senescence may have a major impact on their therapeutic function. This calls for research on senescence and the development of efficient means to rejuvenate MSCs. Recently, several strategies have been explored to rejuvenate senescent MSCs, and subsequently enhance their functions. Overexpression of neuron-derived neurotrophic factor was found to rejuvenate aged BM-MSCs and improve their function in repairing the aged heart after ischemia [82]. microRNA (miR)-10a rejuvenated aged BM-MSCs and enhanced the cardiacprotection following infarction in mice via increased paracrine effects [83]. Furthermore, overexpressing FGF 21 in MSCs may delay their senescence during passaging in vitro [84]. Indeed, rejuvenating MSCs isolated from aged individuals or patients to enhance their functions is of great importance.

### Therapeutic properties of MSCs

Their regenerative and immunomodulatory properties enable MSCs as a novel strategy for treating a wide variety of diseases including autoimmune diseases [85, 86], bone and cartilage diseases [3, 87], cardiovascular diseases [88, 89], inflammatory airway disorders [6, 90], liver diseases [91, 92], muscle diseases [93], neurodegenerative diseases [94, 95], spinal cord injuries [96] and so on. The osteogenic differentiation potential of MSCs makes them successful in treating and managing bone fractures [97]. The ability of MSCs to modulate immune responses is considered as a safe and feasible strategy to treat Crohn’s disease [98], systemic lupus erythematosus (SLE) [99, 100], rheumatoid arthritis (RA) [101], GvHD [102], Type I diabetes [103] and so on. MSCs also prevent allergic airway inflammation and reduce the symptoms of severe asthma [104–108]. Administration of MSCs functionally attenuates airway hyper-responsiveness (AHR), inflammatory cell infiltration, and mucus production in animal models [104, 109–113]. Upon transplantation, MSCs have shown various favourable effects in treating neurodegenerative diseases via enhanced neurogenesis, inflammation modulation, and abnormal protein aggregate clearance [94]. Thus, MSCs have shown promising results in the clinical application of stem cell therapy.

## Clinical application of MSCs

The safety, feasibility and efficacy of MSC therapy for different diseases has been extensively investigated over the past decades. The recent development of MSC-based products for treating diseases provides a bridgehead from which MSCs can be implemented in clinical utility. Considering both the ongoing and completed clinical trials, MSC-based treatment appears to maintain the promise of safety and demonstrates that MSC administration is feasible. However, despite MSC application in the early stage of clinical trials, much work is needed before MSCs can pass from the bench to the bed-side [114]. Table 2 lists some of the clinical trials with outcomes involving the administration of MSCs. Some studies have shown beneficial effects, whereas some studies have shown no effects of MSCs. These mixed and contradictory results in clinical trials hamper the application of MSCs. Among 178 registered clinical trials using umbilical cord-derived MSCs between years 2007–2017, only 16% had status-completed by 27th October, 2018. During the same time, a total of 98 clinical studies were published. Although 74% of the publications reported some promising results, only 18% of the publications showed that this treatment was safe [115]. Although the safety of MSC transplantation was confirmed, less than 40% of the studies and clinical trials with available and published results showed positive improvements in the use of MSCs for patients with amyotrophic lateral sclerosis [116]. Administration of MSCs in clinical trials exhibited beneficial effects on diabetes. However, no significant therapeutic effect was observed and the clinical measures were rapidly restored to the baseline [117]. Compared to adult MSCs, clinical trials using iPSC-MSCs have just begun. The first clinical trial using iPSC-MSCs is now underway to test the clinical efficacy in human patients with steroid-resistant acute GvHD (ClinicalTrials.gov Identifier: NCT02923375). The utilized iPSC-MSCs have been found to be safe and well tolerated in the first cohort (of eight GvHD patients) enrolled in a phase I trial (https://www.cynata.com/graftversushostdisease) [118].

**Table 2:** Summary of some clinical trials with outcomes involving MSC administration

Disease	MSC S ource	Dosage and delivery route	Efficacy	NCT number/reference
Amyotrophic lateral sclerosis	Autologous bone marrow-derived MSCs	1 × 10^6^ cells/kg, via 2 repeated intrathecal injections	Delayed disease progression	NCT01363401
	Autologous adipose-derived MSCs	1 × 10^7^ – 1 × 10^8^ cells, via intrathecal injection	No effect	NCT01609283
	Autologous bone marrow-derived MSCs	15 × 10^6^ cells, via intrathecal injection	Variable effects	NCT02881489
Type 2 diabetes mellitus	Autologous bone marrow-derived MSCs	Injected into the gastroduodenal artery/ pancreaticoduodenal artery	Improvement in daily insulin requirements. Nausea and vomiting were recognized	[119, 120]
	Placental-derived MSCs	1.35 × 10^6^ cells/ kg, 3 intravenous infusions at 1-month intervals	Improvements in C-peptides, HbA1c levels, and insulin dosages. Nausea and vomiting were recognized	[121]
Spinal cord injury	Autologous bone marrow-derived MSCs	8 × 10^6^ cells, via intrathecal administration	Improvement in ASIA score, EMG, and SEP; improvement in MRI imaging	[122]
	Autologous bone marrow-derived MSCs	89.7 × 10^6^ cells, via intra-arterial or intravenous administration	No significant improvement	[123]
	Autologous bone marrow-derived MSCs	1 × 10^6^ cells, via intrathecal administration	Variable patterns of recovery	[124]
	Autologous bone marrow-derived MSCs	7 × 10^5^ to 1.2 × 10^6^ cells, via intrathecal administration	Positive trend, but not statistically significant	[125]
Stroke	Autologous bone marrow-derived MSCs	50–60 × 10^6^ cells, via intravenous administration	No improvement in all clinical scores	[126]
	Autologous bone marrow-derived MSCs	4.57 × 10^7^ MSCs per intravenous infusion were administered amounting to 8.54 × 10^5^ per kilogram body weight at two occasions (4 weeks apart)	Improvements in motor disability and cognitive impairment	[127]
	Umbilical cord-derived MSCs	5 × 10^6^–1 × 10^7^ cells, via intraventricular administration	Safe and feasible	[128]

The contradictory results in MSC clinical application may be caused by the heterogeneity of MSCs, which is the main problem that restricts the therapeutic benefit of MSCs. The heterogeneity of MSCs is influenced by the key parameters of MSCs including donor origin, tissue origin, passage number, expansion protocol, delivery dosage, route and so on. Additionally, multiple factors including the culture condition, the exact diseases intended to be targeted, and the local conditions of administration may also affect the immunomodulatory function of MSCs. These factors directly affect the outcome of MSC-based application. More importantly, many clinical trials have similar limitations in examining the effects of MSCs, including small size, lack of control arms in some cases, and inconsistent methods of isolating and using MSCs. Homogeneity and quality control are the most critical issues for the clinical application of MSCs. Larger studies with more randomized, blinded, strictly-regulated trials and longer follow-up times that show the beneficial effects of MSCs are also needed. This implies that the efforts of researchers and clinicians will focus on revealing the mechanisms that affect the effects of MSCs.

## Mechanisms underlying MSC-based therapy

**Fig. 2:**
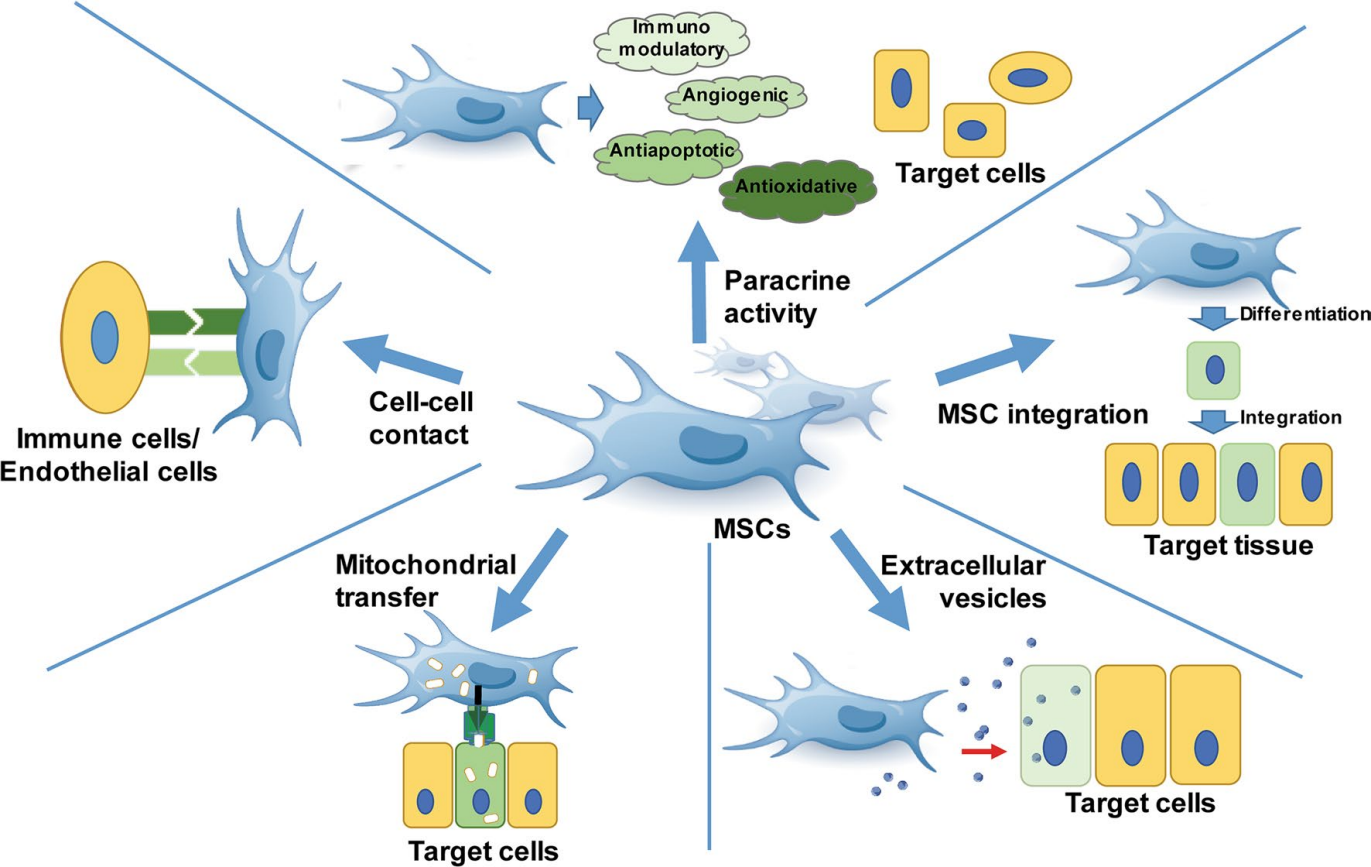
Mechanisms underlying MSC-based therapy. MSCs rescue and/or repair injured cells via differentiation into replacement cell types and by modulating immune responses. The immunomodulatory modes of MSCs include paracrine activity, cell–cell contact and interaction, mitochondrial transfer, and release of extracellular vesicles. The mechanisms involved in repair are not equivalent and MSCs can adapt their therapeutic effects according to diverse local microenvironments. *MSCs* mesenchymal stem cells

The therapeutic potentials of MSCs are mainly attributed to two aspects: first, replacement of the damaged tissue by differentiating into various cell lineages, and the second, regulation of immune responses by immunomodulatory function. Rather than long-term engraftment and differentiation of the integrated MSCs, a growing body of studies has shown that the protective effects of MSCs for damaged and diseased tissues are attributed to alternative immunomodulatory modes. The major mechanism underlying MSC-based therapy is the paracrine function, which secretes a variety of soluble factors to exert immunomodulatory, angiogenic, antiapoptotic and antioxidative effects [129, 130]. Cell–cell contact enables MSCs to modulate their immunosuppressive effects and promote cell viability. MSCs can transfer mitochondria to injured cells via tunnelling nanotubes (TNT) [131, 132]. Furthermore, MSCs reduce inflammation and increase cell proliferation during tissue repair via releasing exosomes that contain reparative peptides/proteins, mRNA, and microRNA (miRNA) (Fig. 2) [133, 134].

### Integration of differentiated MSCs

MSCs have remarkable differentiation potential. After transplantation, differentiated MSCs can successfully integrate into the diseased host tissue. Integration of stem cells is necessary for the improvement of endogenous tissue repair, in order to replace the dead or damaged cells. MSCs and their progenitors can differentiate into chondrocytes and undergo chondrogenesis [135–137]. MSCs can differentiate into cardiomyocyte-like cells, integrate into host tissue, and enhance resident cell activity [138]. With the help of nano-biomaterials, MSCs have achieved better differentiation and functional integration for repairing myocardial infarction repair [139–141]. Transplanted MSCs can integrate into partially hepatectomized or toxic-injured liver for hepatic regeneration [142, 143]. Integration of MSCs has also demonstrated promising results in the treatment of neurodegenerative diseases. MSCs can integrate into the parenchyma of both the brain and the spinal cord. Intraparenchymal delivered MSCs were proven to be safe, and significantly delayed the loss of motor neurons [144]. Tzameret et al. found that intravitreally injected MSCs ameliorate retinal degeneration by integrating into the neural layers of the damaged retina [145]. Moreover, analysis of tissues after MSC transplantation revealed cell fusion between transplanted MSCs and cells of the recipient, albeit at a low frequency. MSC fusion was observed in many organs such as the brain, retina, the liver, muscles, and the gut where they participated in the reestablishment of tissue function [146]. The exact biological implication of MSC fusion is unclear. However, it is worth mentioning that cell fusion between MSCs and cancer cells enhances metastatic capacity and the characteristics of cancer stem cells by undergoing epithelial-mesenchymal transition, which is considered a key cell event in the process of tumour metastasis and invasion [147, 148]. Overall, the engraftment and differentiation efficacy of MSCs post-transplantation is very low which heavily limits their therapeutic effects. The differentiation potential of MSCs largely depends on donor age, tissue origin, cell passage numbers, cell densities, duration of cell culture and so on. Therefore, further investigation is needed to reveal the mechanisms of regulatory pathways and improve differentiation efficacy.

### Soluble factors

#### Immunomodulatory factors

The low immunogenicity makes MSCs eligible for allogeneic transplantation. MSCs can inhibit CD4^+^ cell, CD8^+^ T cell, and NK cell proliferation and DC maturation, suppress plasma cell immunoglobulin production, and stimulate Treg proliferation by secreting transforming growth factor-β (TGF-β), hepatocyte growth factor (HGF), inducible indoleamine 2,3-dioxygenase (IDO), human leukocyte antigen class I molecule (HLA)-G5, prostaglandin E2 (PGE2), interleukin (IL)-6, IL-10, TNFα-stimulated gene protein (TSG)-6 and so on. Bartholomew A. et al. showed that MSCs suppress lymphocyte proliferation, alter lymphocyte reactivity to allogeneic target cells, and prolong skin graft survival following intravenous administration in MHC-mismatched baboons [149]. Furthermore, Di Nicola et al. demonstrated that soluble factors secreted by MSCs partly contribute to immunomodulatory capacity in a co-culture of MSCs with T-lymphocytes in a Transwell system, which excludes cell–cell contact. TGF-β or HGF are involved in the inhibition of T cell proliferation by the addition of a monoclonal antibody in the system [150]. The secreted TGF-β promotes the differentiation of naive T cells into Treg cells, thus improving systemic immune tolerance [151]. Furthermore, Zhong et al. demonstrated that the TGF-β1/Smad signalling pathway is involved in the immunomodulatory effects of MSCs in chronic allergic airway inflammation [152]. MSCs primed with IFN-γ will secrete IDO, which subdues the T-cell response to autoantigens and foetal alloantigens [153]. Furthermore, IDO catalyses the conversion of tryptophan to kynurenine, which inhibits T-cell proliferation [154]. Additionally, the primed MSCs secrete programmed death-ligand 1 (PD-L1), which co-inhibits the adaptive immune response in tissue allografts, autoimmune disease and other diseases [155]. MSC-secreted HLA-G5 suppresses T lymphocytes and NK function, and induces the expansion of CD4^+^CD25^high^FOXP3^+^ Treg cells [71]. MSCs regulate adaptive immune responses by secreting PGE2, which induces DCs to upregulate the anti-inflammatory cytokine IL-10, while reducing the secretion of pro-inflammatory tumour necrosis factor (TNF)-α and IL-12 [156, 157]. This will convert the pro-inflammatory Th1 cells to the anti-inflammatory Th2 cell phenotype. Meanwhile, naïve T cells differentiate into Treg cells, which further decrease the total number of T helper cells [156]. Moreover, MSCs exert immunomodulatory effects by secreting IL-6, which inhibits lymphocytes apoptosis [158]. In addition, MSC-derived nitric oxide (NO) [159], leukaemia inhibitory factor (LIF) [160], galectin-1, and semaphorin-3A [161] inhibit T lymphocyte proliferation. LIF suppresses T cell proliferation as well as promotes and maintains Tregs [162].

MSC-derived soluble factors also modulate macrophage behaviour. The pro-inflammatory phenotype M1 (classical-activated macrophage) transits to the anti-inflammation phenotype M2 (alternative-activated macrophage) in the presence of MSCs [163]. Tsyb et al. demonstrated that MSC-derived TSG-6, via the CD44 receptor, ameliorates macrophages to secrete inflammatory factors by inhibiting NF-κB activity. TSG-6 also inhibits the maturation and function of DCs [164, 165]. Zhang et al. found that galectin-1 from MSCs stimulates the formation of a tolerance immunophenotype on DCs via regulating the MAPK signalling pathway in DCs, thereby inhibiting their function [166]. The immunomodulatory effects of MSC-derived factors have been observed during both the antigen recognition/presentation stage and T cell activation stage of the immune response. Recent studies have shown that the immunosuppressive potency of MSCs is predominantly mediated by key molecules including Rap1 and IDO1 [167, 168]. Furthermore, novel strategies including hypoxia preconditioning and chemical pre-treatment can significantly enhance the immunosuppressive potency of MSCs [169, 170].

MSCs mediate immune responses via diverse modes of action. MSCs can be either immunosuppressive or immune-enhancing depending on the soluble factor levels in the microenvironment. Li et al. found that the degree of NO production acts as a switch in MSC-mediated immunomodulation. MSCs tend to promote T cell proliferation rather than immunosuppression when inducible nitric oxide synthase (iNOS), one of three key enzymes generating NO, is blocked. The level of iNOS/IDO plays a critical role in determining the pathophysiological roles of MSCs [171]. Cuerquis et al. further confirmed that MSCs induce a transient increase in IFN-γ and IL-2 synthesis by activating T cells before suppressing T-cell proliferation [172]. Therefore, in addition to MSC isolation protocols, their origins and dosages, the inflammatory state and level of soluble factors in immune diseases must also be considered before MSC intervention.

#### Angiogenic factors

It has been proven that the angiogenic (the sprouting of existing vessels) and arteriogenic (the growth of collateral vessels) properties of MSCs contribute to the amelioration of hind limb ischemia, coronary artery disease, and skin wound repair [55, 58, 173–176]. Angiogenesis is a complex multistep process that forms new blood networks, which requires endothelial cell growth and differentiation-associated soluble growth factors such as vascular endothelial growth factor (VEGF) and fibroblast growth factor (FGF) [177]. MSCs secrete VEGF, FGF, HGF, placental growth factor (PGF), monocyte chemotactic protein 1 (MCP-1), stromal cell-derived factor 1 (SDF-1), and angiopoietin-1 (Ang-1) that are critical for vascularization [178–183]. Several studies have reported the potential of increasing capillaries and newly formed vessels following MSC administration both in vitro and in vivo [184–186]. Hung et al. demonstrated that angiogenic factors including IL-6, MCP-1, and VEGF in MSC-conditioned medium inhibit apoptosis, increase survival, and stimulate angiogenesis of endothelial cells under hypoxic challenge [179]. IL-6 promotes angiogenesis and survival of endothelial cells [187]. MCP-1 has been proven as a critical chemoattractant for angiogenesis [188]. VEGF can promote MSC differentiation as well as regulate endothelial cell migration, differentiation and endothelialisation via activation of the mitogen-activated protein kinase (MAPK), phosphoinositide-3-kinase and Akt (PI3K/AKT), Src, and Rac pathways [189]. Overexpression of Erb-B2 receptor tyrosine kinase 4 (ERBB4) can rejuvenate aged MSCs and stimulate angiogenesis by regulating the PI3K/AKT and the MAPK/ERK pathways, leading to increased therapeutic effects for myocardial infarction [190]. MSCs promote angiogenesis via the SDF-1/C-X-C chemokine receptor type 4 (CXCR4) axis [191]. Moreover, Dong et al. found that myocardial CXCR4 is required for MSC-derived SDF-1, meditating repair in acute myocardial infarction [192]. MSC-derived angiogenic factors such as SDF-1 and HGF promote local angiogenesis [193, 194]. SDF-1 stimulates endothelial cell proliferation and capillary tube formation, whereas HGF promotes tyrosine phosphorylation in endothelial cells and smooth muscle cells via the c-Met receptor [195, 196]. Further, MSC-derived angiogenic soluble factors improve angiogenesis and restore blood supply in ischemic areas. However, it is unclear whether MSC-derived soluble factors account for the dominant mechanisms of action. The importance of hibernating cells and susceptible cells in the local region should also be considered [129, 197].

#### Anti-apoptotic factors

The multiple roles of apoptosis in regulating various physiological and pathological functions implicate its significance in disease treatment [198]. Moreover, MSCs can synthesise and secrete B-cell lymphoma 2 (BCL-2), survivin, VEGF, HGF, insulin-like growth factor-I (IGF-I), stanniocalcin-1 (STC1), TGF-β, FGF, and granulocyte–macrophage colony-stimulating factor (GM-CSF), which inhibit cellular apoptosis and restore tissue homeostasis [198–203]. BCL-2 is a classic inhibitor of apoptosis. An increased ratio of BCL-2 to BCL-2-associated X protein (BAX) results in cells that are less sensitive to the pathological stimuli and prevents cells from responding to apoptotic signals [204, 205]. Transplantation of autologous MSCs significantly downregulates Bax expression levels in the ischemic myocardium [206]. Zhang et al. demonstrated that the Bcl-2 signalling pathway, together with PI3K/Akt, closely participates in the anti-apoptotic action of MSCs against stroke [207]. Pan et al. demonstrated that MSCs ameliorate hepatic ischemia/reperfusion injuries via inactivation of the MEK/ERK signalling pathway in rats. Meanwhile, MSC-conditioned medium could down-regulate Bax, TNF receptor superfamily, member 6 (FAS), and caspase 3 (CASP3) levels in a human normal liver cell line under ischemic conditions, indicating the anti-apoptotic effects of MSC paracrine function [208]. MSC-derived chemokine (C motif) ligand (XCL1) has been reported to inhibit apoptosis in C2C12 cells [198]. However, direct XCL1 treatment showed no anti-apoptotic capacity.

In addition to the direct inhibition of apoptosis, MSC-secreted factors enhance cell survival by suppressing apoptotic pathways. The levels of VEGF, HGF, IGF-I, FGF, and GM-CSF in MSC culture medium have been found to be significantly elevated under hypoxic conditions [201]. Notably, upregulation of VEGF under hypoxia is greater than that of the other factors [199]. VEGF has been reported to inhibit serum starvation-induced vascular endothelial cell apoptosis via upregulating Bcl-2 expression [209]. VEGF also contributes to suppressing p53-mediated apoptosis via the activating phosphorylation of focal adhesion kinase (FAK), which is essential for regulating cell survival [210, 211].

#### Antioxidative factors

Reactive oxygen species (ROS), including oxygen ions, oxygen-free radicals, and peroxides, are byproducts of normal aerobic metabolism. ROS are involved in the regulation of multiple signalling pathways including cell proliferation, survival, and inflammation [212–214]. An imbalance between levels of ROS and antioxidant function leads to ROS-related diseases such as ageing, carcinogenesis, immune disorders, inflammation, multiple sclerosis, and neurodegeneration [129, 215]. Further, MSCs modulate the redox context via secretion of STC1, heme oxygenase-1 (HO-1), and glial-derived neurotrophic factor (GDNF) [216–218]. MSC-derived STC1 reduces ROS-induced apoptosis. Liu et al. demonstrated that STC1 suppresses angiotensin II-induced superoxide generation in cardiomyocytes via the uncoupling protein 3 (UCP3)-mediated anti-oxidant pathway [219]. Moreover, MSC-derived STC1 enhances the uncoupling respiration of mitochondria, reduces oxidative stress, and promotes the survival of alveolar epithelial cells under harmful microenvironments via upregulation of uncoupling protein 2 (UCP2) [220]. Furthermore, Ono et al. found that STC1 contributes to the ability of MSCs to ameliorate lung fibrosis via inhibition of the ROS/endoplasmic reticulum stress (ER-stress)/TGF-β1 pathway [221]. Oh et al. found that STC1 can also respond to activated macrophages by inhibiting activation of the NLRP3 inflammasome, which decreases mitochondrial ROS production [222]. MSC-derived antioxidative enzyme HO-1 protects against oxidative injury. Allogeneic MSC transplantation ameliorates the redox environment via upregulating HO-1 in a rat model of lipopolysaccharide (LPS)-induced acute lung injury [217]. Chen et al. further demonstrated that HO-1 exerts a protective effect by elevating the activity of nuclear factor-erythroid 2 (NF-E2) p45-related factor-2 (Nrf2), which is a transcription factor mediating the Nrf2-antioxidant response element signalling pathway [223, 224]. HO-1 also attenuates LPS-induced inflammatory and oxidative damage via the enhanced paracrine function of stem cells. Zarjou et al. found that the production of HGF, SDF-1 and VEGF is significantly reduced in HO-1^−/−^ MSCs [225]. MSCs exert localized neuroprotection from oxidative stress by the secretion of GDNF [218, 226]. Lv et al. found that GDNF possibly prevents and repairs neuronal injury by regulating the MEK/ERK and the PI3K/AKT signalling pathways [227]. MSCs secrete different antioxidative factors in different experimental settings and diseases, probably due to the variation of ROS in localized microenvironments.

### Cell–cell contact

MSCs exert their modulatory functions to host cells at damaged sites via paracrine action and direct cell–cell contact. MSCs modulate both autologous and allogeneic T lymphocytes via the expression of integrins (alpha 1 – alpha 6, alpha V, and beta 1 – beta 4), intercellular adhesion molecules (ICAM-1, ICAM-2), vascular cell adhesion protein (VCAM)-1, CD72, and CD58 (LFA-3) on their surfaces [6]. Accumulating evidence has shown that MSCs modulate T cells by the negative costimulatory molecule B7-H4, Fas-L/Fas interaction, or PD-L1/programmed death-1 (PD-1) pathways [228–230]. Kovach et al. demonstrated that the expression of ICAM-1 and VCAM-1 on MSCs is critical for maintaining their immunomodulatory functions on various subtypes of T cells [231]. The expression of PD-1 ligand on the surface of MSCs is critical for the contact-dependent inhibition of allogeneic Th17 differentiation [232]. Galectin-1 and galectin-3 are necessary for MSCs to inhibit the proliferation of CD4^+^ and CD8^+^ T cells [233].

Direct cell–cell contact is required for MSCs to induce Treg cells and in allergic diseases [106, 234]. It has been reported that increased gene expression of the Notch ligand, Delta-like 1, is essential for augmented Treg cell induction by toll-like receptor (TLR)-activated MSCs, which is dependent on cell–cell contact [7]. In addition, MSCs require cell–cell contact to reduce NK-cell cytotoxicity [235]. When co-cultured with MSCs, NK cells acquire CD73 expression, which makes the cells capable of converting adenosine 5′-monophosphate into adenosine for immunomodulatory purposes [236]. Li et al. found that cell–cell contact with pro-inflammatory macrophages enhances TSG-6 production by MSCs, thereby elevating the immunomodulatory effect of MSCs on T cells and macrophages. Pro-inflammatory macrophages in contact with MSCs also upregulate CD200 on stem cells, and skew the reprogramming of macrophages towards an anti-inflammatory phenotype through the interaction of CD200 with CD200R on pro-inflammatory macrophages [237]. Zhang et al. found that MSCs drive mature DCs to differentiate into regulatory DCs via contact-dependent activation of Jagged-2 [238]. Furthermore, direct cell–cell contact between MSCs and endothelial progenitor cells induces MSC differentiation towards a pericyte-like phenotype, which may benefit angiogenesis for cell-based tissue-engineered bone grafts [239]. However, intravenously administered MSCs inhibit endothelial cell proliferation and angiogenesis via cell–cell contact through modulation of the VE-Cadherin/β-catenin signalling pathways [240]. Therefore, the contact-dependent factors affecting the biology of adjacent responder cells and tissues should be carefully considered for optimization of the strategies involving MSCs.

### Mitochondrial transfer

Mitochondria play important roles in the regulation of oxidative phosphorylation, generation of ATP, and cellular apoptosis. Dysfunctional mitochondria lead to excessive ROS production and cause oxidative damage in cells [241]. Accumulating evidence has suggested that mitochondrial transfer from MSCs is a novel strategy for the regeneration of various damaged cells via rescue of their respiratory activities. Accumulating evidence has shown that mitochondrial transfer occurs via TNTs, gap junctions, microvesicles, cell fusion and transfer of isolated mitochondria [132, 242–245]. So far, mitochondrial transfer from MSCs has demonstrated protective effects in lung injury, bronchial epithelial injury, allergic diseases, damaged cardiomyocytes, alkali-burnt corneal epithelial cells, kidney injury, ischemic damage, neurotoxicity, and spinal cord injury [132, 246–253]. Numerous studies have identified several signals including release of damaged mitochondria, mtDNA and mitochondrial products along with elevated ROS levels that trigger mitochondrial transfer from MSCs to the recipient cells [241].

Mitochondrial transfer through TNT has been intensively investigated between MSCs and damaged cells. Miro1 (mitochondrial Rho-GTPase 1, synonym: RhoT1), a calcium-sensitive adaptor protein, has been identified as one of the key regulators in mediating the transport of mitochondria. Miro1 binds the mitochondria to KIF5 motor protein together with other accessory proteins like Miro2, TRAK1, TRAK2, Myo10, and Myo19, thus forming a motor-adaptor complex that coordinates the mitochondrial movement at intercellular and intracellular levels [241, 242]. Knock-down of Miro1 in MSCs inhibits mitochondrial donation, thus reducing their therapeutic effects in bronchial epithelial injury [254]; in contrast, Miro1overexpression in MSCs leads to enhanced beneficial effects [242, 255, 256]. Apart from Miro1, Zhang et al. found that TNF-α induces TNT formation in MSCs via the TNF-α/NF-κB/TNFαIP2 signalling pathway, which facilitates mitochondrial transfer to cardiomyocytes. It has been reported that ROS signals can stimulate TNT formation [251]. Moreover, connexin 43 (CX43) is involved in regulating mitochondrial transfer from MSCs via TNT formation. CX43 overexpression in iPSC-MSCs enhances TNT formations and improves the mitochondrial transfer efficacy between MSCs and damaged epithelial cells. Knock-down of CX43 reduces TNT formation and thus decreases mitochondrial transfer from MSCs to damaged epithelial cells, impairing their immunomodulatory effects during allergic airway inflammation [105].

Additionally, gap junction channels play a critical role in mediating the mitochondrial transfer of MSCs. Islam et al. revealed that MSCs formed CX43-containing gap junction channels with alveolar epithelia in mice with acute lung injury, and released mitochondria-containing microvesicles that were subsequently engulfed by the epithelia. MSCs with genetically modified CX43 failed to adhere to alveolar epithelium and transfer mitochondria [132]. Pacak et al. demonstrated that cardiomyocytes could uptake the mitochondria isolated from MSCs through actin-dependent endocytosis [257]. Sinclair et al. summarized different modes of intercellular communication and mitochondrial transfer by MSCs. Retinoic acid, a gap junction potentiator, greatly enhances the mitochondrial transfer efficiency from BM-MSCs to neurons, and this effect is partially abrogated by 18β glycyrrhetinic acid, which is a gap junction potentiator [253]. Inhibiting microtubule/TNTs, gap junction formation, or microvesicle endocytosis abrogates the transfer of cytoplasmic material from MSCs to epithelial cells [258]. Notably, MSCs can donate mitochondria to macrophages via extracellular vesicles, thus promoting an anti-inflammatory macrophage phenotype in acute respiratory distress syndrome [259]. Different pathophysiological conditions may initialize different modes of mitochondrial transfer, though their potential mechanisms remain unclear. Therefore, clarifying the relative mechanisms involved in mitochondrial transfer will advance the understanding of molecules involved in this process and serve to improve MSC treatment.

### Extracellular vesicles (exosomes)

Extracellular vesicles (EVs), the membrane-bound vesicles released by somatic cell, are involved in tissue repair, immunomodulation, and proliferation [260–262]. EVs are classified into exosomes (30–150 nm endosome-derived plasma membrane-coated vesicles), microvesicles (100–1000 nm non-endocytic origin vesicles) and apoptotic bodies (1–5 μm vesicles released by apoptotic cells) according to their size and biogenesis. The most common EV markers are ALG-2-interacting protein X (Alix), tetraspanin proteins CD9, CD63, CD81 and heat-shock protein (Hsp)60, Hsp70, and Hsp90. In addition, MSC-released EVs express unique surface antigens including CD44, CD73, CD90 and CD105 [263].

EVs, especially exosomes purified from MSCs have attracted great attention due to their regenerative, immunomodulatory, and even anti-tumour properties. Over the past decade, MSC-EVs have been found to exhibit various biological effects and have emerged as a novel approach for treating a variety of diseases. They overcome some limitations of MSC-based therapies including allogeneic immune rejection, malignant transformation, and premature cell differentiation. EVs have the unique capability to cross the blood–brain barrier, which is very important in the treatment of neurological disorders [263]. This means that EVs have better advantages in the clinic in the treatment of nervous system diseases as compared to the therapeutic potential of MSCs. Moreover, MSC-EVs can avoid the risk of genetic changes associated with stem cell transplantation for the treatment of nerve disorders [264, 265]. Remarkably, MSC-EVs can be modified to carry specific proteins or genes that promote cellular function and tissue repair. These characteristics make the EVs an ideal candidate of treatment for regenerative medicine.

MSC-EVs enhance angiogenesis owing to their specific protein and transcript contents related to angiogenic and proliferative function [266, 267]. Anderson et al. further demonstrated that the protein content in MSC exosomes mediates angiogenesis via regulation of the NF-κB signalling pathway [267]. Nakamura et al. reported that MSC-derived exosomes promote muscle regeneration by enhancing angiogenesis and myogenesis, which is partially mediated by miR-494 [268]. Feng et al. demonstrated that miR-22 in MSC exosomes prevents apoptosis and reduces the infarct size in the heart by targeting methyl CpG binding protein 2 (Mecp2) [269]. In addition, miR-223 in MSC-EVs is involved in mediating cardioprotection via targeting semaphorin-3A (Sema3A) and transcription 3 (Stat3) [270]. miR-19a contributes to the anti-apoptotic effects of MSC exosomes in cardioprotection [271]. MSC exosomal miRNAs (miR-21, miR-23a, miR-125b and miR-145) contribute to the suppression of myofibroblast formation by inhibiting TGF-β2/Smad2 signalling and reducing scar formation during wound healing [272]. Tomasoni et al. reported that MSC exosomes improve renal cell survival and proliferation by transferring the mRNA for insulin-like growth factor 1 receptor (IGF-1R), which increases the sensitization of proximal tubular cells to IGF-1 [273]. Currently, several strategies are under exploration that aim to enhance the exosomes released from MSCs. Hypoxia can facilitate MSCs to release exosomes, thus improving repair of cardiac tissues in a mouse model of myocardial infarction [274]. Compared with MSCs, exosomes derived from SDF1-overexpressing MSCs show enhanced therapeutic effects in myocardial infarction by increasing cardiac endothelial microvascular regeneration and inhibiting cardiomyocyte apoptosis in mice [275].

MSC-EVs modulate the immune system by induction of anti-inflammatory cytokines and Treg cells, by inhibition of B lymphocytes, regulation of macrophage polarization, and mobilization of neutrophils [260, 276]. Zhang et al. found that MSC-derived exosomes induce monocytes to differentiate into macrophages via the myeloid differentiation primary response gene 88 (MYD88)-dependent TLR signalling pathway. Exosome-induced macrophages lead to Treg cell expansion by secretion of more IL-10 as compared to the macrophages induced by lipopolysaccharide [134]. miR-146a enhances macrophage polarization to anti-inflammatory M2 macrophages [271]. Di Trapani et al. further demonstrated that the immunosuppressive effect of EVs on T cells, B cells, and NK cells is also mediated by PD-L1 expression on their surface [277]. Additionally, Galectin-1, an endogenous leptin on the EV surface, was also found to be involved in the immunosuppressive effects on T lymphocytes [278]. Kerkela et al. also emphasized the importance of 5′-ectonucleotidase (CD73), which actively produces immunosuppressive adenosine [279].

MSC-EVs have shown positive outcomes in treating cancer. Anti-angiogenic miRNAs such as miR-16 and miR-100 have been identified in MSC exosomes, which suppress angiogenesis by targeting VEGF in breast cancer cells [133, 280]. However, the crosstalk between MSCs and tumour cells through EVs can function either as a tumour suppressor or as a promoter [281, 282]. MSC exosomes may transfer CD73 on tumour cells, which can reduce activation of NK cell and T cell by metabolism of AMP to adenosine [283]. So far, MSC-derived exosomes have been reported to be involved in tumour growth, angiogenesis, metastasis, and invasion [284]. The discrepancy between these controversial behaviours may arise from issues related to different MSC sources, tumour types, stages of tumour growth, and genotypes. Therefore, the potential side effects of EV therapy must be carefully evaluated.

### The target cell profile

One of the major mechanisms underlying MSC-based therapy is interaction with target cells. MSCs modulate their immunomodulatory effects by suppressing the proliferation and activity of T cells, promoting Treg cells, regulatory DCs and M2 macrophages in a myriad of inflammatory diseases [285]. In case of T cell suppression, Lin et al. examined the mRNA expression profiles in mouse T lymphocytes after MSC administration and found that 5 mRNAs including *Ccl11*, *Ccl24*, *Il13*, *Il33*, and *Ear11* were significantly altered [109]. Wang et al. further identified more than 800 differentially expressed long non-coding RNAs (lncRNAs) in mouse T lymphocytes, and lncRNAs *MM9LINCRNAEXON12105*+ and *AK089315* were finally identified as potential targets of MSC treatment in T cells [107]. MSCs and Treg cells have been found to work and interact in a synergistic manner. Engela et al. demonstrated that Treg cells can induce IDO secretion in MSCs, which results in TNF-α reduction and induction of IL-10 in Treg cells and effector cells [286]. Different subtypes of Treg cells generated by MSCs have been identified including CD4^+^CD25^+^Foxp3^+^ Treg cells and IL-10 producing type 1 Treg (Tr1) cells [287]. There is a complex cross-talk between MSCs and macrophages, which cannot be simply explained by MSC-derived anti-inflammatory factors. Braza et al. found that macrophages can phagocytose MSCs and alter their pro-inflammatory signature to M2 suppressive phenotype following contact with dead MSCs [288]. This behaviour may explain the profound long-term effects of MSC therapy. Therefore, the presence of MSCs alters the targeted cell profile, which in turn leads to further activation or ‘licensing’ of MSC therapy.

## Challenges in MSC-based therapy

MSC-based therapies have made great progress over the last decades. However, the publications/clinical trials with mixed and contradictory results are preventing the advancement of MSCs into daily clinical application. These disparities are probably due to the large variability in key factors such as cell source (tissue, donor), dosage, administration route, and administration timing. Inconsistencies among these parameters significantly limit the therapeutic value of MSCs. Therefore, standardization of procedures of MSC isolation and expansion is crucial for upcoming clinical therapeutics. The in vivo administration route, timing, and dosage also require optimization. In this circumstance, an understanding of the characteristics and functional mechanisms of differently sourced MSCs is required. The therapeutic benefits of MSCs are contributed by their differentiation potential and immunomodulatory capacity. These potentials are strongly influenced by the tissue source of MSCs, the age and health condition of the donor or the ex vivo culture conditions before administration. Furthermore, the indications of the local disease microenvironment where MSCs are intended to be applied also determine the benefits of MSCs. As a result, preconditioning strategies are developed that boost the differentiation or immunomodulatory potential of MSCs in such scenarios. Hypoxic preconditioning is employed since physiological environments are often hypoxic, and MSCs cultured under such condition show enhanced viability and secretion of cytoprotective molecules. However, slight variations in the oxygen level may significantly influence the function of MSCs as they are highly sensitive to oxygen tension [289]. Preconditioning with cytokines such as IFN-γ or TNF-α enhances immunomodulatory factor secretion by MSCs, but such effects have been reported as temporary [69, 290]. Alternative tissue engineering approaches including three-dimensional culture and hydrogel encapsulation were employed to enhance MSC functions [291, 292]. The therapeutic potentials of MSCs are attributed to complex cellular and molecular mechanisms of action, and such mechanisms still require in-depth exploration for clinical application. Current researches have made great progress and are gaining advancements in enhancing the therapeutic properties of MSCs and creating specific criteria to establish the basics for clinical application of MSCs. Moreover, senescence of MSCs has also attracted significant attention during the past years. MSCs can only undergo very limited cell passages and prolonged expansion, inevitably leading to replicative senescence. MSCs isolated from aged individuals or from patients also exhibit a senescent phenotype and display decreased function.

## Conclusion

The advantages of MSCs in immunomodulation and tissue repair have rendered the cells an important source for stem cell therapies. The potential and eligibility of allogeneic cells makes MSCs desirable for cellular transplantation. Based on the promising results in preclinical and clinical studies, the emerging commercially available MSC-based products have been approved globally. However, larger studies with more randomized, blinded, and controlled trials are desired to demonstrate the beneficial effects of MSCs. This implies that the mechanisms underlying MSC-based therapy should be addressed. So far, MSCs have been intensively investigated for their differentiation capacity, paracrine effects, flexible EV release, and direct-contact modulatory functions. Each mechanism contributes to the comprehensive process of MSC therapy. Nevertheless, mechanisms underlying the protective effects of MSCs still require further elucidation. MSCs can adapt therapeutic effects during the rescue and repair of damaged tissues according to diverse local microenvironments. Therefore, the in-depth mechanisms underlying the protective effects of MSCs require further investigation. Clarification of the predominant mechanisms in different situations will improve the safety, efficacy and outcomes of MSC-based therapy.

## Abbreviations

AHR: Airway hyper-responsiveness
Alix: ALG-2-interacting protein X
Ang-1: Angiopoietin-1
ASCs: Adipose-derived stem cells
BAX: BCL-2-associated X protein
BCL-2: B-cell lymphoma 2
CASP3: Caspase 3
CX43: Connexin 43
CXCR4: Chemokine receptor type 4
DC: Dendritic cell
ER: Endoplasmic reticulum
ERBB4: Erb-B2 receptor tyrosine kinase 4
EVs: Extracellular vesicles
FAK: Focal adhesion kinase
FGF: Fibroblast growth factor
GM-CSF: Granulocyte–macrophage colony-stimulating factor
GDNF: Glial-derived neurotrophic factor
GvHD: Graft-versus-host disease
hESCs: Human embryonic stem cells
HGF: Hepatocyte growth factor
HLA: Human leukocyte antigen
HLA-G5: Human leukocyte antigen class I molecule G5
HO-1: Heme oxygenase-1
Hsp: Heat-shock protein
ICAM: Intercellular adhesion molecules
IDO: Inducible indoleamine 2,3-dioxygenase
IFN: Interferon
IGF-I: Insulin-like growth factor-I
IGF-1R: Insulin-like growth factor 1 receptor
IL: Interleukin
ILCs: Innate lymphoid cells
iNOS: Inducible nitric oxide synthase
iPSCs: Induced pluripotent stem cells
ISCT: International Society of Cell Therapy
LIF: Leukemia inhibitory factor
lncRNAs: Long non-coding RNAs
LPS: Lipopolysaccharide
MAPK: Mitogen-activated protein kinase
MCP-1: Monocyte chemotactic protein 1
Mecp2: Methyl CpG binding protein 2
Miro1: Mitochondrial Rho-GTPase 1
miRNA: MicroRNA
MSCs: Mesenchymal stem cells
MYD88: Myeloid differentiation primary response gene 88
NK: Natural killer
NO: Nitric oxide
Nrf2: Nuclear factor-erythroid 2 p45-related factor 2
PBMCs: Peripheral blood mononuclear cells
PD-1: Programmed death-1
PD-L1: Programmed death-ligand 1
PGE2: Prostaglandin E2
PGF: Placental growth factor
PI3K/AKT: Phosphoinositide-3-kinase and Akt
RA: Rheumatoid arthritis
ROS: Reactive oxygen species
SDF-1: Stromal cell-derived factor 1
Sema3A: Semaphorin-3A
SLE: Systemic lupus erythematosus
STC1: Stanniocalcin-1
TGF-β: Transforming growth factor-β
Th: T-helper
TLR: Toll-like receptor
TNF: Tumour necrosis factor
TNTs: Tunnelling nanotubes
Tr1: Type 1 Treg
Tregs: Regulatory T cells
TSG: TNFα-stimulated gene protein
UCP: Uncoupling protein
VCAM: Vascular cell adhesion protein
VEGF: Vascular endothelial growth factor
XCL1: Chemokine (C motif) ligand
